# ﻿A crane fly of the genus *Gynoplistia* Macquart (Diptera, Limoniidae) from the early Miocene of New Zealand

**DOI:** 10.3897/zookeys.1192.115536

**Published:** 2024-02-20

**Authors:** André Nel, Uwe Kaulfuss

**Affiliations:** 1 Institut de Systématique, Évolution, Biodiversité (ISYEB), UMR 7205, Muséum national d’Histoire naturelle, CNRS, Sorbonne Université, EPHE, Université des Antilles, 75005 Paris, France Sorbonne Université Paris France; 2 Georg-August-University, Department of Animal Evolution and Biodiversity, Untere Karspüle 2, 37073 Göttingen, Germany Georg-August-University Göttingen Germany

**Keywords:** Australasia, Fossil-Lagerstätte, Foulden Maar, Insecta, Tipuloidea

## Abstract

The first fossil limoniid fly from the Miocene Fossil-Lagerstätte of Foulden Maar in New Zealand is described on the basis of an isolated well-preserved wing. The specimen is tentatively attributed to a new species *Gynoplistiafouldensensis***sp. nov.** in the large extant genus *Gynoplistia*, which is well diversified in the country. It is the second fossil record of this genus, the first one being an isolated wing from the Cretaceous Weald Clay Formation in the United Kingdom.

## ﻿Introduction

Limoniid flies are very frequent in the fossil record, with 468 species distributed in 48 genera (Alroy 1998). They are supposed to be among the oldest known Diptera, with a fossil record dating back to the Triassic (Krzemiński and Krzemińska 2003; Kopeć et al. 2020). However, they remain quite difficult to study because of the lack of information on the body structures in many fossils. Nevertheless, many Cretaceous and Cenozoic fossils are attributed to extant genera, suggesting an impressive morphological stability through time for the whole family.

The fossil limoniids from Australasia are very poorly known, with two “limoniid indet.” briefly described and figured by Jell and Duncan (1986: figs 49, 50) and Jell (2004: figured on p. 104), one undescribed Miocene record (McCurry et al. 2022), and one Upper Jurassic genus and species described to date from Australia (Oberprieler et al. 2015). Thus, each new fossil is welcome to increase our knowledge on the past history of these flies in this region.

Limoniids are frequently encountered in the Miocene lacustrine sediments and amber from Europe, China, Russia, Sumatra, Mexico, and Dominican Republic (e.g., Gentilini 1984; Wu et al. 2019; Ngô-Muller et al. 2021).

Here we describe a new limoniid species based on an isolated wing from the early Miocene of New Zealand, we tentatively attribute it to the genus *Gynoplistia* Macquart, 1835. With 319 extant species, this genus is very speciose and distributed all over the world (Oosterbroek 2024). Nevertheless, the only previously fossil known was *Gynoplistia* (?) *mitchelli* Jarzembowski, 1991, described on the basis of an isolated wing from the Early Cretaceous of the United Kingdom (Jarzembowski 1991).

## ﻿Materials and methods

**Figure 1.**
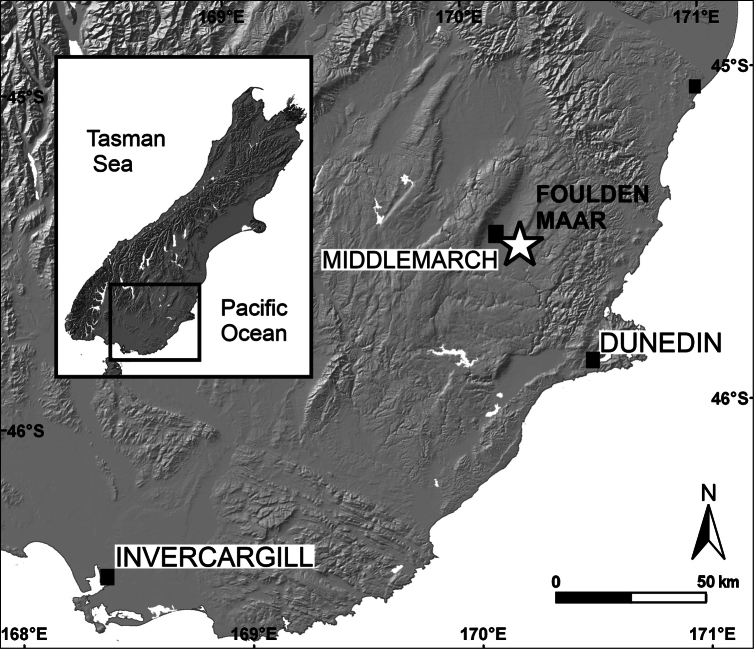
Map of the South Island of New Zealand showing the location of the Foulden Maar fossil site.

The single specimen described herein was collected at the Foulden Maar Fossil-Lagerstätte (Fig. 1) near Middlemarch, Otago, southern New Zealand (45.5269°S, 170.2191°E) in a diatomite mining pit, which is registered as I43/f8503 in the New Zealand Fossil Record File (GNS Science and Geoscience Society of New Zealand 2003). The varved and highly fossiliferous diatomite at the fossil site represents the latest uppermost *Rhoipiteswaimumuensis* (Couper, 1923) to lower early *Proteaciditesisopogiformis* Couper, 1960 pollen zones, corresponding to New Zealand local stages late Waitakian-early Otaian (earliest Miocene, Aquitanian) (Mildenhall et al. 2014). Geological setting and palaeoecology of the fossil site are summarised by Lindqvist and Lee (2009), Lee et al. (2016, 2022), and Kaulfuss (2017).

The specimen was studied and photographed with a Nikon SMZ1000 stereomicroscope with attached Canon T3 camera. Wetting the specimen with ethanol revealed venational details of the wing and enhanced the contrast between the diatomite matrix and the fossil.

Photographs were stacked and enhanced in Photoshop CS5.1 (Adobe Systems Inc.) and the drawing of the wing was prepared from photographs using CorelDraw. We follow the wing venation terminology of de Jong (2017).

Wing nomenclature: CuA, cubitus anterior; CuP, cubitus posterior; A, anal vein; d, discal medial cell; M_1_, M_2_, M_3_, M_4,_ branches of median vein; m1, cell between M_1_ and M_2_; Rs, posterior branch of radius; R_1_, R_2_, R_3_, R_4_, R_5,_ apical branches of radius; r-m, crossvein between R_5_ and M_1+2_; Sc, subcostal vein.

## ﻿Systematic palaeontology

**Order Diptera Linnaeus, 1758**

**Family Limoniidae Rondani, 1856**

**Genus *Gynoplistia* Macquart, 1835**

### ﻿ Gynoplistia fouldensensis sp. nov.

Taxon classificationAnimaliaDipteraLimoniidae

003E7C60-B174-5F92-904B-1E30B59E3E2D

https://zoobank.org/E23FB30B-A2E1-477E-B47C-DC875723A096

Fig. 2

#### Type material.

***Holotype***: New Zealand • sex unknown; an isolated wing; near Middlemarch, Otago; Foulden Maar Fossil-Lagerstätte; 45.5269°S, 170.2191°E; Geology Museum, Department of Geology, University of Otago (OU); OU46615.

#### Locality and horizon.

Foulden Maar diatomite, near Middlemarch, Otago, New Zealand; earliest Miocene, Aquitanian.

#### Diagnosis.

The wing venation of the new species strongly resembles that of the fossil *G.* (?) *mitchelli* in the shape of the radial and median veins. Still, *G.fouldensensis* sp. nov. but can be differentiated by the shape of discal cell and crossvein between M_3_ and M_4_ being more distal than basal part of M_3_.

#### Description.

Wing 8.8 mm long, 3.2 mm wide, with brown tinge, a series of white spots in anterior part and five series of transverse darker spots, veins black; Sc long, ending into C, extending far distal beyond fork of Rs, Sc-r just before tip of Sc; part of R_5_ basal to r-m elongate and oblique, R_5_ straight, reaching wing apex, 1.5 as long as Rs, R_2+3+4_ 0.9 mm long; R_2_ beyond fork of R_3_ and R_4_; R_3_ 3.0 mm long, slightly undulate; R_4_ 3.4 mm long, straight; no supernumerary crossveins in cells r3, r4, and r5; r-m and m-cu not aligned, r-m situated a short distance past base of discal medial cell, m-cu situated midway between base and apex of discal medial cell; fork of vein M_3+4_ in apical section of discal medial cell; discal medial cell 1.4 mm long, 0.7 mm wide, closed; cell m1 present, c. 1.2 mm long; vein CuA straight; anal vein straight.

**Figure 2.**
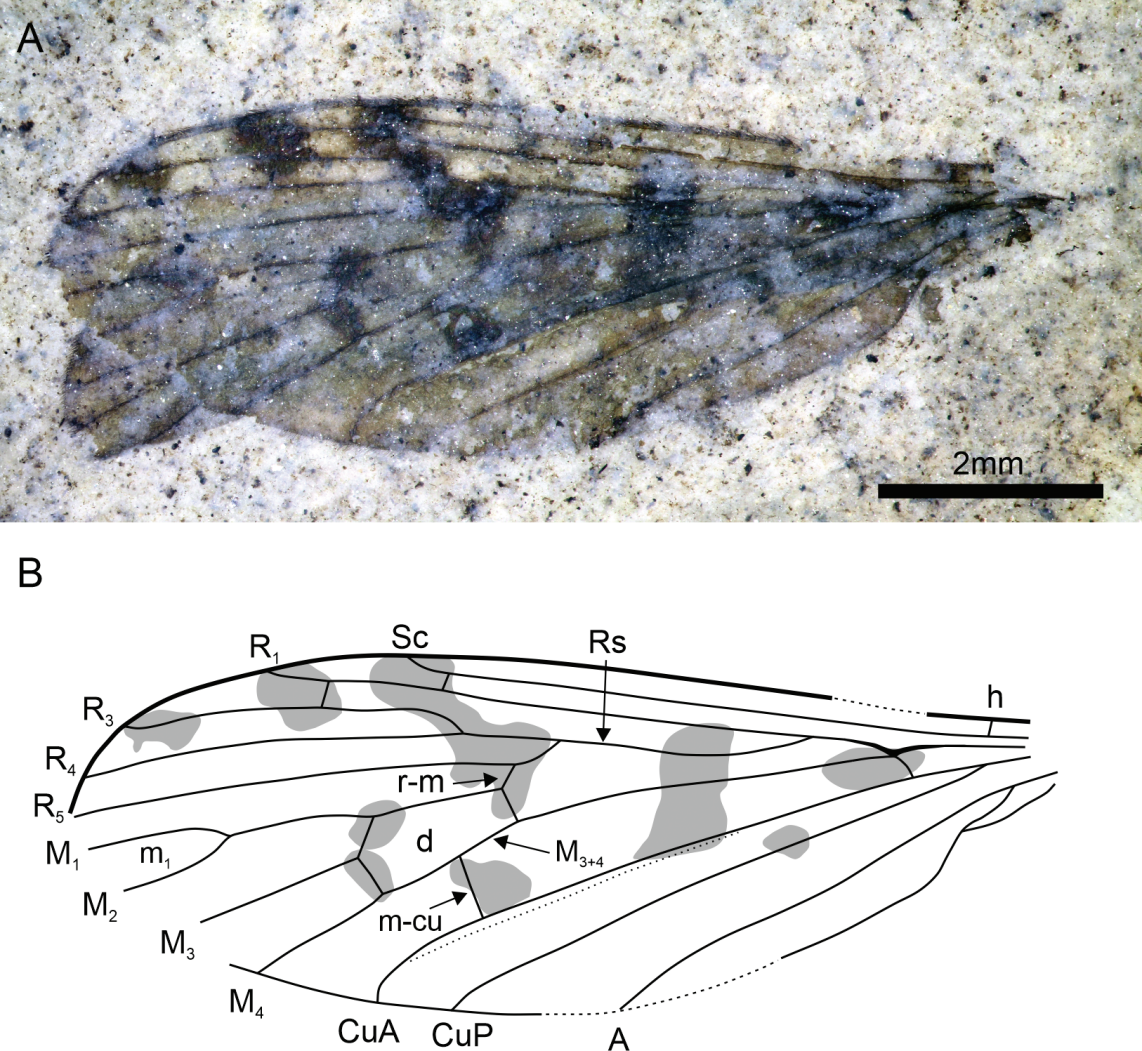
*Gynoplistiafouldensensis* sp. nov., holotype, OU46615 **A** wing photograph **B** interpretative drawing of wing. Scale bar: 2 mm.

#### Etymology.

Named after the type locality Foulden Maar (Otago, New Zealand).

#### Discussion.

This wing corresponds to that of a Limoniidae because of the following characters (after de Jong 2017): well-developed CuP and anal vein; anal vein nearly straight; apex of vein Sc well developed; apices of R_1_ and R_3_ well separated; fork of vein M_3+4_ in apical section of discal medial cell; crossvein m-cu far removed from fork of M_3+4_; vein CuA straight. It is quite delicate to attribute an isolated fossil wing of Limoniidae to a genus because many genera are separated on the basis of body characters.

The combination of characters “cell m1 present, part of R_5_ basal to r-m elongate and oblique, and forked R_2+3+4_” is encountered in some species of the genera *Gynoplistia*, *Pseudolimnophila* Alexander, 1919, *Hexatoma* Latreille, 1809 (*sensu lato*), and *Pilaria* Sintenis, 1889. The Australasian species of *Epiphragma* Osten Sacken, 1860 also have a cell m1 and a forked R_2+3+4_, but their part of R_5_ basad r-m is very short, unlike in the new fossil.

*Hexatoma* (*sensu lato*) forms a morphology-based phylogenetic clade with *Pseudolimnophila*, *Pilaria*, and *Ulomorpha* (Ribeiro 2008).

*Pseudolimnophila* and *Ulomorpha* are unknown in the Australasian/Oceanian region. *Pilaria* is represented by *P.brooksi* Alexander, 1953 in this region. This species has no cell m1 (Alexander 1953).

*Hexatoma* is currently divided into six subgenera (Podenas et al. 2022). The new fossil would fall in the subgenus Eriocera Macquart, 1838 because of the following characters: radial sector with three branches, medial cell distal, supernumerary crossveins missing in cells r3, r4, and r5, vein Sc reaching wing margin beyond Rs branching point, R_2_ beyond fork of R_3_ and R_4_ (Podenas et al. 2022).

Following Oosterbroek (2024), *Hexatoma* is represented in the Australasian region only by five species of the subgenus Eriocera, which are Hexatoma (Eriocera) aperta (Alexander, 1920), H. (E.) atra (Doleschall, 1859), H. (E.) australiensis (Alexander, 1920), H. (E.) metallica (Schiner, 1868), and H. (E.) setifera (Alexander, 1931). The new fossil differs from all these species in the presence of cell m1. Also, H. (Eriocera) metallica differs from the new fossil in the uniformly infuscate wing and aligned r-m and m-cu (Billingham and Theischinger 2022). Hexatoma (E.) australiensis also has wings with a “pale brown suffusion”, and R_2+3+4_ “equal to or a little shorter than R_3_ alone” versus much shorter in the new fossil (Alexander 1920: 104). Hexatoma (E.) aperta has “brownish gray” wings and H. (E.) setifera a blackish tinge, and both have an opened discal medial cell (Alexander 1920: 105, 1931: 166). Hexatoma (E.) atra has R_3_ only slightly longer than R_2+3+4_, and m-cu is situated close to base of the discal medial cell (Edwards 1921). Thus, the new fossil is not similar to any of these species.

Unlike the genera previously mentioned, *Gynoplistia* is very diverse in New Zealand, with 108 species listed by Oosterbroek (2024). Some representatives of this genus have patterns of wing coloration with colored bands and spots, very close to that of *G.fouldensensis* sp. nov. Theischinger (1993) proposed a revision of the Australian species of *Gynoplistia*. Affinities with the subgenus Cerozodia Westwood, 1835 are excluded because of the vein Sc ending into C in the new fossil. The wing venation would rather fit with that of a species of the subgenera *Xenolimnophila* Alexander, 1922 or *Gynoplistia* for the narrow elongate cell r3, the oblique basal part of R_5_, the presence of cell m1, the vein m-cu not aligned with r-m, and the wing coloration with spots and bands (Theischinger 1993: figs 9b, 11b). The New Zealand species of *Gynoplistia* also have wing coloration with spots and bands, but many have a basal part of R_5_ clearly less oblique than in the new fossil (e.g., Edwards 1923: pl. 30; Alexander 1939: pl. 28, fig. 1).

It is noteworthy that the wing venation of the fossil *G.* (?) *mitchelli* strongly resembles that of *G.fouldensensis* sp. nov., especially in the shape of the radial and median veins, but with an important difference in the shape of the discal cell, that is, the crossvein between M_3_ and M_4_ is more distal than basal part of M_3_ in the new fossil versus the contrary in *G.* (?) *mitchelli* (Jarzembowski 1991: fig. 14). Indeed, the discal cell of *G.* (?) *mitchelli* resembles that of the H. (Eriocera) spp.

## ﻿Conclusion

This study of a new fossil wing illustrates the difficulties encountered when describing a fossil Limoniidae on the sole basis of wing characters. In this case at least two genera could be candidates for an attribution, even if we prefer the genus *Gynoplistia* rather than *Hexatoma* mostly because of the pattern of wing coloration. Also the attribution of this Miocene fossil species to to *Gynoplistia* is unsurprising because this genus is nowadays very diverse in New Zealand, whereas *Hexatoma* remains unknown from this country.

## Supplementary Material

XML Treatment for Gynoplistia fouldensensis

## ﻿Additional information

### Author ORCIDs

André Nel https://orcid.org/0000-0002-4241-7651

Uwe Kaulfuss https://orcid.org/0009-0007-6858-8612

### Data availability

All of the data that support the findings of this study are available in the main text.
